# Supplementary material for: Experiences with Glofitamab Administration following CAR T Therapy in Patients with Relapsed Mantle Cell Lymphoma
Source: Cells. 2022 Sep 2;11(17):2747. doi: 10.3390/cells11172747 (PMC9454987; doi:10.3390/cells11172747)
Supplement: Supplementary file 1 [file cells-11-02747-s001.zip › cells-1839468-supplementary.pdf]

Online supplement to:

Experiences with glofitamab administration following CAR T therapy in patients with relapsed mantle cell lymphoma.

[1]Alexander D. Heini, [2]Ulrike Bacher, [2]Naomi Porret, [2]Gertrud Wiedemann, [2]Myriam Legros, [2]Denise Stalder Zeerleder, [1]Katja Seipel, [1]Urban Novak, [2]Michael Daskalakis, and [1]Thomas Pabst

[1] Department of Medical Oncology, Inselspital, Bern University Hospital
[2] Department of Hematology and Central Hematology Laboratory, Inselspital, Bern University Hospital, Bern, Switzerland
*Correspondence: Thomas Pabst, MD; Extraordinary Professor; Department of Medical Oncology; University Hospital and University of Bern; and Center for Hemato-Oncology; University Cancer Center; 3010 Berne; Switzerland. E-Mail: thomas.pabst@insel.ch

Contains two supplemental tables and one supplemental figure.

Pre/Post CAR T (days)	-50	-40	-6		6	15	36	43	115		169	188	209	223	237	258	296
Pre/Post Glofitamab (days)	-212	-202	-168		-156	-147	-126	-119	-47		7	26	47	61	75	96	134
**Leukocytes (10^9/L)**	1.73	2.76	40.3		0.25	1.71	2.37	4.5	2.42		0.68	2.84	2.38	6.4	2.34	1.48	1.22
**Absolute Lymphocyte count (10^9/L)**	0.14	0.22	0.19		0.03	0.05	0.11	0.14	0.17		0.2	0.25	0.22	0.18	0.25	0.18	0.08
**B Cells (% of Lymphocytes)**	0	0	0	**CAR T**	0	0	0.1	0	0	**Glofitamab**	0	0	0.1	0	0	0	0
**T Cells (% of Lymphocytes)**	81	84	58		75	83	29	21	28		40	32	18	24	43	42	34
**CD4+ T cells (% of Lymphocytes)**	51	49	34		37	42	20	15	19		31	27	15	14	27	31	27
**CD8+ T cells (% of Lymphocytes)**	20	24	22		38	41	7	8	7		8	7	3	8	15	14	9
**CD4/CD8 Ratio**	2.55	2.04	1.55		0.97	1.02	2.86	1.88	2.71		3.88	3.86	5	1.75	1.8	2.21	3
**NK cells (% of Lymphocytes)**	18	13	30		18	17	53	63	58		55	54	52	61	39	35	42

Table S1: Flow cytometric assessments of lymphocyte subsets of patient #1. Percentages not adding up to 100% are due to rounding or NK/T cells, which are not reported reliably.

Pre/Post CAR T (days)	-105	-41	-6		5	12	26	40	58	96	117	159	183
Pre/Post Glofitamab (days)	-295	-231	-196		-185	-178	-164	-150	-132	-94	-73	-31	-7
**Leukocytes (10^9/L)**	5.69	4.2	7.97		8.69	9.41	4.39	2.6	2.69	3.04	2.98	2.75	4.28
**Absolute Lymphocyte count (10^9/L)**	2	0.37	0.36		0.09	0.2	0.35	0.19	0.25	0.24	0.25	0.19	0.15
**B Cells (% of Lymphocytes)**	68	0	0	**CAR T**	0	0.3	0	0	0	0	0	0	0
**T Cells (% of Lymphocytes)**	18	74	69		59	46	57	55	49	50	48	40	51
**CD4+ T cells (% of Lymphocytes)**	14	23	29		17	16	25	31	30	32	31	24	35
**CD8+ T cells (% of Lymphocytes)**	5	51	43		44	31	34	26	22	20	18	15	16
**CD4/CD8 Ratio**	2.8	0.45	0.67		0.39	0.52	0.74	1.19	1.36	1.6	1.72	1.6	2.19
**NK cells (% of Lymphocytes)**	14	25	31		42	51	44	46	53	51	50	58	46

Table S2: Flow cytometric assessments of lymphocyte subsets of patient #2. Due to the patient declining further active treatment, no further flow cytometric assessments were made. Percentages not adding up to 100% are due to rounding or NK/T cells, which are not reported reliably.

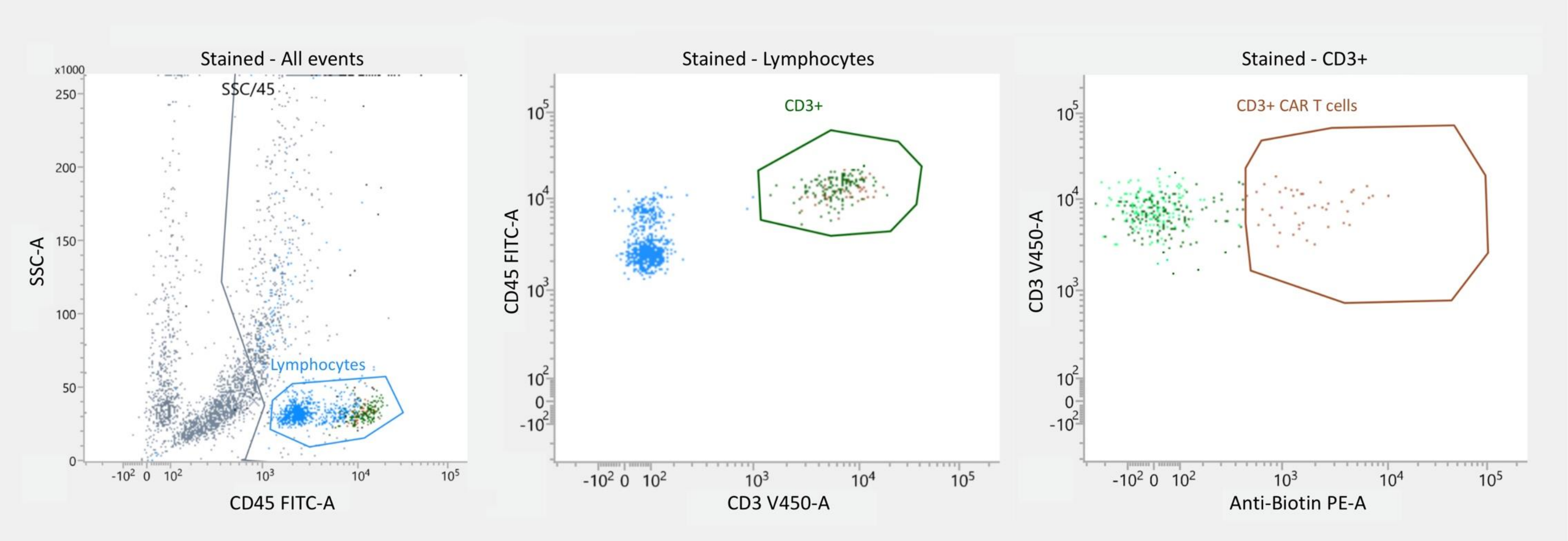

Figure S1: Flow cytometric detection of CAR T cells in cerebrospinal fluid of patient #1 during relapse. The left panel shows a bivariant plot of CD45 against side scatter (SSC) allowing the isolation of lymphocytes (blue gate). The middle panel shows a plot of CD3 against CD45 for the isolation of CD3+ lymphocytes (green gate). The right panel shows the detection of CAR T cells using the CD19 CAR Detection Reagent (Biotin), that detects CD19 CAR T cells via recognition of the CD19 CAR molecule, and that can be identified with flexible Anti-Biotin fluochromes.
